# Fluoride concentration in toothpaste marketed to children in Brazil and Mexico, and discussion on current regulations

**DOI:** 10.1590/0103-6440202204522

**Published:** 2022-04-29

**Authors:** Ademir Melo Leite, Astrid Carolina Valdivia-Tapia, Ritta de Cássia Nascimento Pinto Costa, Emilia Valenzuela Espinoza, Antônio Pedro Ricomini, Jaime Aparecido Cury

**Affiliations:** 1 Piracicaba Dental School, UNICAMP, Piracicaba, SP, Brazil; 2 Federal University of Maranhão, UFMA, São Luís, MA, Brazil; 3 Universidad Nacional Autónoma de Mexico, UNAM, Ciudad de Mexico, Mexico

**Keywords:** Fluoride, dentifrice, toothpaste, dental caries, anti-caries

## Abstract

Fluoride toothpastes market to children should contain a minimum concentration of 1000 ppm of fluoride (F), which must be chemically soluble to provide anti-caries effect. Therefore, we determined the concentrations of total fluoride (TF) and total soluble fluoride (TSF) in toothpastes marketed to children in Brazil and Mexico and analyzed the current regulations in force in both countries. Twenty-four brands were found and purchased in Brazil (19 formulated with NaF/SiO_2_, three with Na_2_FPO_3_/CaCO_3_ and two with Na_2_FPO_3_/SiO_2_) and six in Mexico (all with NaF/SiO_2_). TF and TSF concentrations were determined after the purchase (fresh samples) but fluoride stability in Na_2_FPO_3_/CaCO_3_-formulations was checked after 18 months. The analyses were performed with an ion-specific electrode and the results expressed in ppm F (mg F/kg). The TF concentrations found ranged from 476.0 to 1385.3 ppm F and they were close the declared by the manufactures (500 to 1450 ppm F). The TF concentrations found were not greater than 1500 ppm F, in accordance with the current regulations of both countries. However, toothpastes presenting TSF concentrations lower than 1000 ppm F were found either in low fluoride toothpaste (500 ppm F) formulated with NaF/SiO_2_ as in fresh and aged Na_2_FPO_3_/CaCO_3_-toothpastes, originally fabricated with 1000-1100 ppm of TF. In conclusion, although most toothpastes analyzed showed TSF concentration higher than 1000 ppm F, the regulations in force in both countries allow that products not in agreement with the best available evidence are available in the market.

## Resumo

Os dentifrícios fluoretados comercializados para crianças devem conter concentração mínima de 1000 ppm de fluoreto (F), que deve ser quimicamente solúvel para ter efeito anticárie. Portanto, determinamos as concentrações de fluoreto total (FT) e fluoreto solúvel total (FST) em dentifrícios comercializados para crianças no Brasil e no México e analisamos as regulamentações vigentes em ambos os países. Vinte e quatro marcas foram encontradas e adquiridas no Brasil (19 formuladas com NaF/SiO_2_, três com Na_2_FPO_3_/CaCO_3_ e duas com Na_2_FPO_3_/SiO_2_) e seis no México (todas com NaF/SiO_2_). As concentrações de FT e FST foram determinadas após a compra (amostras frescas), e a estabilidade do fluoreto nas formulações de Na_2_FPO_3_/CaCO_3_ foi verificada após 18 meses. As análises foram realizadas com eletrodo íon-específico e os resultados expressos em ppm F (mg F/kg). As concentrações de FT encontradas variaram de 476,0 a 1.385,3 ppm F e foram próximas às declaradas pelos fabricantes (500 a 1.450 ppm F). As concentrações de FT encontradas não ultrapassaram 1.500 ppm F, de acordo com as regulamentações vigentes de ambos os países. No entanto, dentifrícios com concentrações de FST inferiores a 1.000 ppm F foram encontrados tanto em dentifrício com baixa concentração (500 ppm F) formulado com NaF/SiO_2_ como em dentifrícios com Na2FPO3/CaCO3 frescos e envelhecidos, originalmente fabricados com 1.000-1.100 ppm de FT. Em conclusão, embora a maioria dos dentifrícios analisados apresentasse concentração de FST superior a 1.000 ppm F, as regulamentações vigentes em ambos os países permitem que produtos que não estejam de acordo com a melhor evidência disponível estejam presentes no mercado.

## Introduction

The use of fluoride toothpaste has contributed to control dental caries worldwide ^1^ and its benefit is strongly based on scientific evidence ^2^. Furthermore, the use of fluoride toothpaste since the eruption of the first tooth in the child's mouth has been recommended ^3^ because the anticaries benefit can overcome the risk of dental fluorosis ^4^^,^^5^. For the balance between caries prevention and fluorosis risk, it has been recommended that toothpastes for children should contain from 1000 to 1500 ppm of total fluoride (TF). However, in most countries, toothpastes are considered as cosmetic products and the governmental regulations establish the maximum fluoride concentration allowed but not a minimum ^6^^,^^7^^,^^8^. In addition, most regulations do not establish how much of the TF should be chemically soluble (TSF) in the formulation to guarantee the anticaries effect ^9^.

Therefore, toothpastes marketed to children with TSF lower than 1000 ppm F have been found in the market not because the formulation presents fluoride chemically unstable ^10^^,^^11^ but mainly because is allowed by the national regulations. The toothpastes marketed for children are usually formulated with sodium fluoride (NaF) and silica (SiO_2_) as an abrasive ^11^^,^^12^. As SiO_2_ is an inert component, all F will be soluble, available as ion F (F^-^). However, it possible to find toothpastes formulated with sodium monofluorophosphate (Na_2_FPO_3_) and calcium carbonate (CaCO_3_) as abrasive. In this formulation, soluble F is mainly found as monofluorophosphate ion (FPO_3_^2-^). Although FPO_3_^2-^ is stable in the formulation, it is susceptible to undergo hydrolysis over time, which favors reaction of F^-^ with the calcium from the abrasive, forming insoluble F salts ^13^^,^^14^. Therefore, a reduced F concentration is even more critical for a Na_2_FPO_3_/CaCO_3_-based toothpaste since the soluble F content will be increasingly reduced over time. Thus, while a 500 ppm F toothpaste formulated with silica is able to maintain all TF as TSF overtime, one formulated with abrasive containing Ca as CaCO_3_^9^^,^^15^ or CaH_2_PO_4_.2H_2_O will have lower TSF before the expire date of the product.

Thus, the Brazilian ^6^ and Mexican ^16^ regulations only establish the maximum concentration of TF present in a toothpaste, which should not exceed 0.15% expressed in F (1,500 ppm F; mg F/kg). In addition, the regulations do not mention the minimum concentration of soluble F that a toothpaste should contain and maintain until its expiration date. Therefore, it is mandatory that the regulations be revised in order that the fluoride dentifrices offered to the population, including the ones market for children, provide the anti-caries benefit based on the best evidence available.

Although there is great commercial interest in toothpastes marketed to children, there is no recent study evaluating the fluoride content of these toothpastes sold in Brazil and Mexico. Therefore, the aim of this study was to evaluate the concentrations of total fluoride and total soluble fluoride in toothpastes marketed to children in Brazil and Mexico, and the stability of soluble fluoride concentration in Na_2_FPO_3_/CaCO_3_ formulations. In addition, the current regulations on fluoride dentifrice in both countries were analyzed.

## Methodology

### Sampling

The toothpastes marketed to children were purchased in Brazil and Mexico, and the information found on the packaging is described in Table 1. Twenty-four brands were found in the Brazilian market (Table 1; Codes A to X), being purchased in drugstores in in the cities of Piracicaba, Campinas, and Limeira, SP state. In Mexico, the six brands found were purchased in three supermarkets in Mexico City (Table 1; Codes Y to D1). Toothpaste tubes of different lots (n = 2-3) were used to determine the fluoride content in each formulation. All dentifrices were purchased between September and October in 2019, and the samples were analyzed shortly after the purchase (fresh samples). In April 2021, only Na_2_FPO_3_/CaCO_3_-based toothpastes were re-evaluated (aged samples) to estimate the stability of fluoride in these formulations. Fluoride stability in the other toothpastes was not checked because NaF or NA_2_FPO_3_ are compatible with silica.

**Table 1:** Dentifrices analyzed in the study and information provided by the manufacturers.

Code	Commercial name	Country	n	Formulation	TF declared	Lots	Expiration date
A	Bambinos 2; (2-5 years)	Brazil	2	NaF/SiO_2_	500	L000397; L000550	Jun/2021; Feb/2022
B	G.U.M.; the Lion Guard	Brazil	2	NaF/SiO_2_	995	17539; 17431	Dec/2020; Dec/2020
C	Paw Patrol; (3+ years)	Brazil	3	NaF/SiO_2_	1000	19114; 19086; 19114	Apr/2022; Mar/2022; Apr/2022
D	Sorriso Kids	Brazil	3	NaF/SiO_2_	1100	9144BR122I; 9115BR123C; 9144BR122I	May/2021; Apr/2021; May/2021
E	Tandy	Brazil	3	NaF/SiO_2_	1100	9148BR121C; 9172BR122K ; 9011BR122K	May/2022; Jun/2022; Jan/2022
F	Colgate Smile; (6+ years)	Brazil	3	NaF/SiO_2_	1100	(L)8247MX1136; (L)7285MX1136; (L)9038MX1116	Sep/2021; Oct/2021; Feb/2022
G	Oral-B Kids	Brazil	3	NaF/SiO_2_	1100	83024354P0; 83284354P2; 82704354P0	Sep/2020; Oct/2020; Aug/2020
H	Oral-B Stages	Brazil	3	NaF/SiO_2_	1100	91784354Q1; 81514354Q2; 81274354Q2	May/2021; Apr/2021; Apr/2021
I	Neutrocare	Brazil	3	NaF/SiO_2_	1100	1921; 1914; 1802	Feb/2021; Jan/2021; Oct/2020
J	Peppa Pig; (5+ years)	Brazil	2	NaF/SiO_2_	1100	48347; 44416	May/2022; Oct/2021
K	Dentalclean; (3+ years)	Brazil	3	NaF/SiO_2_	1100	43691; 43542	Sep/2021; Dec/2021
L	Dentalclean; (5+ years)	Brazil	3	NaF/SiO_2_	1100	43691; 43542	Sep/2021; Dec/2021
M	Boni Kids	Brazil	3	NaF/SiO_2_	1100	26106; 13633; L1050078	Dec/2020; Mar/2020; Sep/2021
N	Hello Kitty	Brazil	2	NaF/SiO_2_	1100	27229; 27351	Jan/2021; Feb/2021
O	BITUFO Cocoricó	Brazil	3	NaF/SiO_2_	1100	L8214AS; L8281AS; L8173AS	Aug/2021; Oct/2021; Jun/2021
P	Malvatrikids; F-infantil	Brazil	2	NaF/SiO_2_	1100	181413; 190077	Nov/2021; Feb/2022
Q	Kid's CREST	Brazil	2	NaF/SiO_2_	1100	7334GC; 7334GC	Oct/2020; Oct/2020
R	Bambinos 3; (6+ years)	Brazil	3	NaF/SiO_2_	1100	L000559; L000469; L000613	Mar/2022; Oct/2021; Jun/2022
S	Dentil Kids; Scooby-Doo	Brazil	3	NaF/SiO_2_	1100	38516; 44527; 28867	Nov/2020; Oct/2021; Apr/2021
T	Dora a Aventureira	Brazil	2	Na_2_PO_3_/CaCO_3_	900	11856119; 11856119	Jun/2022; Jun/2022
U	Dentil Kids Zoo	Brazil	2	Na_2_FPO_3_/SiO_2_	1000	A99011; A99011	Jan/2022; Jan/2022
V	Dentil Kids	Brazil	2	Na_2_FPO_3_/SiO_2_	1100	1370018; 28867	Jun/2022; Apr/2021
W	Tra Lá Lá Kids; antiaçúcar	Brazil	3	Na_2_FPO_3_/SiO_2_	1100	L290455; L290369; L290441	Apr/2022; Apr/2021; Mar/2022
X	Tra Lá Lá Kids	Brazil	2	Na_2_FPO_3_/SiO_2_	1179	L290376; L290456	May/2021; Apr/2022
Y	G.U.M.; the Lion Guard	Mexico	3	NaF/SiO_2_	995	17430; 17535; 17535	Aug/2020; Nov/2020; Nov/2020
Z	Colgate Kids	Mexico	3	NaF/SiO_2_	1085	8313BR122k; 8324BR121K; 8286BR122K	Nov/2021; Nov/2021; Oct/2021
A1	Colgate Smile	Mexico	3	NaF/SiO_2_	1100	19023 MX1126; 18362 MX1116; 8292 MX1136	Jan/2022; Dec/2021; Oct/2020
B1	Oral-B Kids	Mexico	3	NaF/SiO_2_	1100	90274354P0; 90334354P0; 83554354P2	Dec/2020; Jan/2021; Nov/2020
C1	Oral-B Stages	Mexico	3	NaF/SiO_2_	1100	83614354Q0; 83304354Q3; 83624354Q0	Nov/2021; Oct/2021; Nov/2021
D1	Crest (Star Wars)	Mexico	2	NaF/SiO_2_	1450	72694354P1; 72694354P1	Aug/2020; Aug/2020

TF declared = Total fluoride declared by the manufacturer on label; ppm F = mg F/kg; NaF = sodium fluoride; Na_2_FPO_3_ = sodium monofluorophosphate ; SiO_2_= silica; CaCO_3_ = calcium carbonate.

### Determination of Fluoride Concentration

The concentrations of total fluoride (TF) and total soluble fluoride (TSF) in toothpastes were determined for all formulations (Na_2_FPO_3_/CaCO_3_ and NaF/SiO_2_). Fluoride concentration as MFP ion (FPO_3_^2-^), fluoride ion (FI) and the percentage of insoluble fluoride (% ins-F) was calculated only for Na_2_FPO_3_/CaCO_3_-based toothpastes. Depending on the formulation, Na_2_FPO_3_/CaCO_3_ or NaF/SiO_2_, dentifrice samples were differently prepared.

For Na_2_FPO_3_-based toothpastes, fluoride analysis was carried out following the conventional protocol, as previously described by Cury et al., ^17^. Briefly, from 90 to 110 mg of toothpaste was weighed (± 0.01 mg), and then the dentifrice sample was vigorously homogenized in 10.0 mL of purified water. Duplicates of 0.25 mL of the toothpaste slurry were transferred to test tubes for TF concentration analysis. The remaining suspension was centrifuged (3,000 *g* for 10 min at room temperature) and duplicates of 0.25 mL of the supernatant were transferred to test tubes test for TSF and FI determination. To TF and TSF tubes, 0.25 mL of 2 M HCl was added, and the samples were incubated for 1 h at 45ºC (water bath) to hydrolyze the FPO_3_^2-^ ion. Then, the samples were neutralized with 0.5 mL of 1 M NaOH and buffered with 1.0 mL of TISAB II. For FI analysis, duplicates of 0.25 mL of the supernatant were transferred to tubes test, and 0.5 mL of 1 M NaOH, 1.0 mL of TISAB II, and 0.25 mL of 2 M HCl were added in this order to avoid FPO_3_^2-^ ion hydrolysis. From these analyses, the concentrations of F as FPO_3_^2-^ ion (FPO_3_^2-^ = TSF - FI) and the percentage of ins-F were calculated [(TF found - TSF) x 100/(TF found)].

For the NaF-based toothpastes, a validated simplified protocol was used ^18^ discarded the unnecessary steps of acid hydrolysis. The same amount of toothpaste was weighed and homogenized in purified water. Duplicates of 1.0 mL of the toothpaste slurry were transferred to test tubes for TF analysis. After centrifugation, duplicates of 1.0 mL of the supernatant were transferred to tubes for TSF determination. TF and TSF were buffered with 1.0 mL of TISAB II.

### Fluoride analysis

Fluoride analysis was carried out using an ion specific electrode (9609BNWP, Thermo Scientific Orion, Cambridge, MA, USA) coupled to an ion analyzer Versa Star (Thermo Scientific Orion, Cambridge, MA, USA). A calibration curve was made with F standards of different concentration prepared as the samples. For Na_2_FPO_3_-based toothpastes, standards ranged from 0.0625 to 2.5 μg F/mL in 0.25 M HCl, 0.25 M NaOH, and TISAB II 50% (v/v). For NaF-based toothpastes, standards ranged from 0.5 to 10.0 μg F/mL in TISAB II 50% (v/v). The accuracy of the analysis was checked with a standard fluoride solution (Orion 940907, Thermo Scientific) and the average coefficient of variation from triplicates was 0.4% and 0.2%, respectively for Na_2_FPO_3_ and the NaF calibration curves. Results were expressed as ppm F (mg F/Kg).

### Legislations about fluoride toothpastes

To analyze the legislations on fluoride toothpastes in both countries, a search was performed on the websites of Brazilian Ministry of Health and Mexican Official Journal of the Federation. The Table 2 describes the main data obtained, including the maximum fluoride allowed in the toothpaste formulation.

**Table 2:** Specifications about Brazilian and Mexican resolutions on fluoride dentifrices

Country	Resolution/Standard	Year	Supervisory entity	Classification	Fluoride concentration
Brazil ^6^	Resolution number 79, ANVISA	2000	ANVISA (Brazilian Health Surveillance Agency)	Cosmetic product	1,500 ppm (0.15%) of total fluoride as the maximum concentration allowed
Mexico ^16^	Standard NMX-K-539-NYCE-2020	2020	Normalización y Certificación NYCE, S.C.	Cosmetic product	1,500 ppm (0.15%) of total fluoride (as ion fluor) as the maximum concentration allowed

## Results

In Brazilian sampling, a total of 24 different brands of dentifrices marketed to children were found, while in Mexico, only 6 brands (Table 1). Moreover, in Brazil, it was possible to observe different formulations, being 19 (79%) of the toothpastes formulated with NaF/SiO_2_, 3 (13%) with Na_2_FPO_3_/CaCO_3,_ and 2 (8%) with Na_2_FPO_3_/SiO_2._ Different from the Brazilian diversity, in Mexico all the 6 toothpastes were formulated with NaF/SiO_2._

Considering the total F declared by the manufacturers (Table 1), most of toothpastes, 3 from Mexico and 18 from Brazil in a total of 21 (70%), were formulated with 1100 ppm F. The other six formulations in Brazil contained 500 (A), 900 (T), 995 (B), 1000 (C, U), and 1179 (X) ppm F. In Mexico, the other three formulations disclosed 995 (Y), 1085 (Z) and 1450 (D1) ppm F.

**Figure 1:**
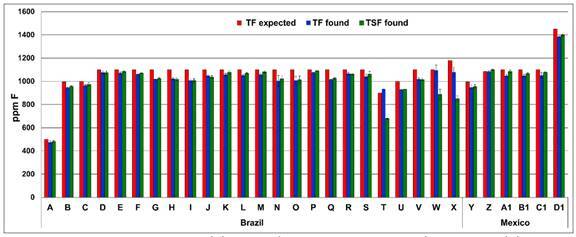
Concentrations of total fluoride (TF) expected (declared by the manufacturer), total fluoride (TF) and total soluble fluoride (TSF) in toothpastes purchased in Brazil and in Mexico (ppm F). Analyses were performed in Sept-Oct 2019 with fresh samples.

The TF concentration found in all products ranged from 476.0 to 1385.3 ppm F, being in accordance with what was declared by the manufacture (Figure 1). All concentrations were also in accordance with Brazilian and Mexican regulations, presenting a TF concentration that did not exceed 1.500 ppm F (Table 2).

The TSF concentration in fresh samples of NaF-based toothpastes ranged from 480.7 to1398.8, while for Na_2_FPO_3_-based toothpastes, from 677.4 to 1015.7 (Figure 1). Most of toothpastes marketed to children in Brazil, 17 (70.9%), and in Mexico, 5 (83.4%), had a concentration of soluble F greater than 1,000 ppm, ranging from 1005.1 to 1110.5 in Brazilians and from 1066.9 to 1398.8 in Mexicans.

Na_2_FPO_3_-based toothpastes presented lower TSF concentration than TF found (Table 3) when CaCO_3_ was the abrasive used in the formulation (T, W, X), which was not observed for the silica-containing (U, V). Fresh samples of the Na_2_FPO_3_-based toothpastes T, W, and **X** presented 27.4, 19.0 and 21.2% of insoluble fluoride, respectively, with TSF concentration lower than 886.5 ppm F.

**Figure 2:**
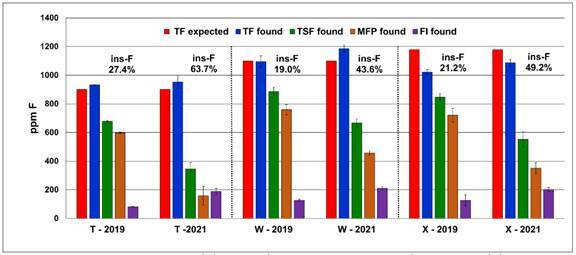
Concentrations of total fluoride (TF) expected (declared by the manufacturer), total fluoride (TF), total soluble fluoride (TSF), FPO_3_^2-^ ion, fluoride ion (FI) and percentage of insoluble fluoride (% ins-F) found in Na_2_FPO_3_/CaCO_3_-based toothpastes fresh (2019) and after 18-month storage at room temperature (2021).

The aged samples of Na_2_FPO_3_/CaCO_3_ toothpastes presented an increased amount of insoluble fluoride after the 18-month period (Figure 2). The aged samples of toothpastes T, W, and **X** presented 63.7, 43.6, and 49.2% of insoluble fluoride, respectively, with TSF concentration lower than 667.7 ppm F, reaching to 345.8 ppm F in toothpaste T.

## Discussion

In this study, the concentrations of total fluoride (TF) and total soluble fluoride (TSF) present in toothpastes marketed to children in Brazil and Mexico were evaluated. In Brazil, the fluoride concentration declared by the manufactures ranged from 500 to 1,179, while in Mexico from 995 to 1,450 ppm F. All the toothpastes presented TF concentration similarly to the F content described by the manufacturer. Among the toothpastes formulated with SiO_2_ as abrasive (NaF/SiO_2_ and Na_2_FPO_3_/SiO_2_), the TSF concentration was similar to the TF concentration found. On the other hand, the toothpastes formulated with Na_2_FPO_3_ and CaCO_3_ as abrasive presented lower TSF concentration compared to the TF concentration. In these brands, the % of insoluble fluoride increased from 27.4 % in fresh samples to 63.7% after 18 months of storage. Considering all the dentifrices evaluated, 70.9% of the Brazilians and 83.4% of the Mexicans presented a TSF concentration greater than 1,000 ppm F, the minimum concentration necessary to provide anti-caries effect.

In Brazil, it was found a great diversity of brands and formulations of dentifrices marketed to children when compared to Mexico (Table 1). While in Mexico, the available brands are from multinational companies, in Brazil 70% of the toothpastes evaluated were manufactured by small local producers. All the Mexican toothpastes and most of the Brazilian presented NaF/SiO_2_ formulations, showing TSF concentration similar to the TF found (Figure 1). Toothpastes having SiO_2_ as abrasive, allows to prepare gel formulations with different colors and flavors, being attractive to children. Differently from NaF-based, Na_2_FPO_3_-based toothpastes were only found in the Brazilian market. The use of Na_2_FPO_3_ in a formulation is necessary when CaCO_3_ is used as abrasive, to avoid F reaction with the calcium from the abrasive, forming insoluble F salts ^13^^,^^14^^,^^19^. Na_2_FPO_3_/CaCO_3_-based toothpastes have a lower cost of production and have an affordable price compared to SiO_2_/NaF-based formulations, having a social impact for developing countries.

Among the Na_2_FPO_3_-based toothpastes found in Brazil, two was formulated with Na_2_FPO_3_/SiO_2_ (codes U and V), showing the TF and TSF concentrations were similar (Table 3). As expected, SiO_2_ as an inert abrasive does not interfere with the soluble fluoride content. However, the dentifrices formulated with Na_2_FPO_3_/CaCO_3_ (codes T, W and X) presented 19.0% to 27.4% of insoluble fluoride (Table 3) in fresh samples, which shows a considerable reduction of TSF concentration when compared to the TF found. Although these formulations had declared from 900 to 1,179 ppm F, the TSF concentration found ranged from 677.4 to 886.5 ppm F. After an 18-months period, the Na_2_FPO_3_/CaCO_3_ dentifrices (codes T, W and X) presented 43.6% to 63.7% of insoluble fluoride (Figure 2), which represents around 50% of the F content in the formulation. The prolonged storage period favored the continuous formation of insoluble F salts ^13^^,^^14^^,^^19^.

**Table 3:** Total fluoride (TF) concentration expected, TF and total soluble fluoride (TSF), soluble fluoride found as monofluorophosphate ion (FPO_3_^2-^) and F ion (FI), and percentage of insoluble F (%Ins-F) in fresh samples of Na_2_FPO_3_-based toothpastes purchased in Brazil.

Code	TF expected	ppm F found as		ppm F found as		%Ins-F
		TF	TSF	FPO_3_^2-^	FI	
T	900	932.7 ± 0.6	677.4 ± 3.5	597.0 ± 5.5	80.4 ± 2.1	27.4 ± 0.4
U	1000	928.5 ± 0.0	932.2 ± 0.0	904.4 ± 0.2	28.8 ± 1.2	0.4 ± 0.0
V	1100	1019.7 ± 10.5	1015.7 ± 10.4	985.4 ± 9.2	30.4 ± 1.2	0.4 ± 0.0
W	1100	1094.7 ± 46.2	886.5 ± 44.6	759.7 ± 66.4	126.8 ± 21.8	19.0 ± 0.7
X	1179	1078.8 ± 40.8	850.5 ± 25.5	720.4 ± 35.9	126.5 ± 8.1	21.2 ± 4.2

Regardless of the target audience, a toothpaste must contain a minimum concentration of 1,000 ppm of soluble F to provide anti-caries effect ^20^^,^^21^. An interesting result was that most toothpastes were formulated with 1100 ppm F (Table 1), which could favor the minimum concentration necessary to be effective. However, we still found in the Brazilian market a toothpaste with low fluoride concentration (A). Another concern is related to Na_2_FPO_3_/CaCO_3_ toothpastes containing around 1,000 ppm F, since the soluble fluoride content is reduced in recently acquired toothpastes, with the concentration decreasing over time. Therefore, it would be necessary to increase the concentration of TF in the formulation, considering that part of fluoride would be insoluble. This already occurs in Na_2_FPO_3_/CaCO_3_ toothpastes sold for the general public, since these formulations usually contain 1450/1500 ppm F. Another solution would be for companies to invest in the development of more stable Na_2_FPO_3_/CaCO_3_ formulations that do not compromise the soluble fluoride content, providing an affordable and anti-caries effective formulation for the entire population.

All the evaluated dentifrices presented TF concentration lower than 1,500 ppm F, being in accordance with the current Brazilian ^6^ and Mexican ^16^ regulations that establishes that the maximum concentration of fluoride in dentifrices should not exceed 1,500 ppm F. Different from the Brazilian, the Mexican standard declares that the concentration refers to fluoride ion, however, the presence of FPO_3_^2-^ ion present in Na_2_FPO_3_-based toothpastes, marketed to the general public, is also a soluble fluoride source that should be included in the Mexican standard. Unfortunately, the current Brazilian resolution, which is similar to Mercorsul ^7^ and European Union ^8^, and Mexico standard do not specify how much of soluble F should be present in a toothpaste formulation to be anti-caries effective. Previous studies have pointed out the need of change in the Brazilian regulation ^9^^,^^22^, however, it remains unchanged. Therefore, the regulations of both countries must establish the minimum concentration of soluble F that a toothpaste should contain and maintain to provide anti-caries effect to the entire population, irrespective of age.

The lack of regulations that state a minimum concentration of soluble F in toothpastes impacts not only Brazil and Mexico, being a worldwide problem signaled by the World Dental Federation ^23^. FDI advocates the use of toothpaste with a fluoride concentration between 1,000 to 1,500 ppm, with a minimum of 800 ppm of soluble fluoride. The concentration of 800 ppm F can be justified since Na_2_FPO_3_/CaCO_3_ toothpaste would hardily maintain a concentration higher than 1000 ppm F close to the expiration date. Therefore, it is expected that Na_2_FPO_3_/CaCO_3_ formulations containing 1450/1500 ppm F, present the minimum concentration of 1,000 ppm of soluble F in recently acquired toothpastes (fresh sample), and the concentration of 800 ppm F after two years from product manufacture (aged sample), that could be a feasible concentration to be obtained even by small producers.

The absence of updated regulations establishing the minimum concentration of F in dentifrices enables that dentifrices with low F concentration continue to be recommended. Like the Mexican guideline for prevention and control of oral diseases ^24^, in which the use of toothpaste containing 550 ppm F is recommended for children under 6 years old, and also informs that toothpaste with a concentration of 551 to 1,500 ppm F should only be used by children over 6 years old. Despite of this recommendation, low fluoride dentifrices (< 600 ppm F) were not found in the largest supermarkets of Mexico City. The use of low-F formulations has been raised as an alternative to reduce the risk of fluorosis. However, the use of low-F dentifrices by preschoolers did not reduce the risk of caries in the primary dentition and did not decrease the risk of fluorosis in permanent teeth ^21^. Therefore, fluoride toothpastes with conventional concentration (1000 to 1500 ppm F) should be recommended to children, using an age-related amount of toothpaste for tooth brushing till age of six ^25^.

In conclusion, toothpastes marketed to children in Brazil and Mexico are diverse in terms of brand, formulation, and the fluoride content. All toothpastes presented TF concentration lower than 1500 ppm F, being in accordance with current regulations of both countries. Most of the Brazilians and Mexicans toothpastes presented TSF concentration greater than 1000 ppm F, the minimum concentration necessary to provide anti-caries effect. Na_2_FPO_3_/CaCO_3_ toothpastes presented reduced TSF concentration, highlighting the need for improvements in formulations. In addition, the regulations of both countries should be revised, requiring the minimum concentration of 1,000 ppm of soluble fluoride to provide anti-caries effect not only to children, but also to the entire population.
